# Material Removal Mechanism of SiC Ceramic by Porous Diamond Grinding Wheel Using Discrete Element Simulation

**DOI:** 10.3390/ma17112688

**Published:** 2024-06-02

**Authors:** Zhaoqin Zhang, Jiaxuan Xu, Yejun Zhu, Zhongxing Zhang, Weiqi Zeng

**Affiliations:** College of Engineering, Nanjing Agricultural University, Nanjing 210031, China; 9213011329@stu.njau.edu.cn (Z.Z.); xujiaxuan@stu.njau.edu.cn (J.X.); 15970311437@163.com (Z.Z.); zengwq@stu.njau.edu.cn (W.Z.)

**Keywords:** porous diamond, DEM, SiC, self-sharpening, grinding

## Abstract

SiC ceramics are typically hard and brittle materials. Serious surface/subsurface damage occurs during the grinding process due to the poor self-sharpening ability of monocrystalline diamond grits. Nevertheless, recent findings have demonstrated that porous diamond grits can achieve high-efficiency and low-damage machining. However, research on the removal mechanism of porous diamond grit while grinding SiC ceramic materials is still in the bottleneck stage. A discrete element simulation model of the porous diamond grit while grinding SiC ceramics was established to optimize the grinding parameters (e.g., grinding wheel speed, undeformed chip thickness) and pore parameters (e.g., cutting edge density) of the porous diamond grit. The influence of these above parameters on the removal and damage of SiC ceramics was explored from a microscopic perspective, comparing with monocrystalline diamond grit. The results show that porous diamond grits cause less damage to SiC ceramics and have better grinding performance than monocrystalline diamond grits. In addition, the optimal cutting edge density and undeformed chip thickness should be controlled at 1–3 and 1–2 um, respectively, and the grinding wheel speed should be greater than 80 m/s. The research results lay a scientific foundation for the efficient and low-damage grinding of hard and brittle materials represented by SiC ceramics, exhibiting theoretical significance and practical value.

## 1. Introduction

SiC ceramics are hard and brittle materials with high mechanical strength, excellent chemical stability, a small thermal expansion coefficient, and high resistance to wear and corrosion. They have been widely applied in aero-engine components and satellite optical mirrors [1,2]. In general, diamond grinding wheels, composed of monocrystalline diamond grit (M-diamond), serve as the main method for the manufacturing of SiC ceramics. Though this grit type has superior mechanical properties, macro-fractures still occur [3,4,5]. The grit type seriously affects the service life of the grinding wheel and produces a large amount of irreversible surface/subsurface damage on the SiC surface as the wheel wears [6]. Conventional M-diamond grits cannot satisfy the requirements of the high-efficiency and low-damage processing of SiC ceramics. Thus, porous diamond grits (P-diamond) have been developed [7,8]. Moreover, numerous microporous structures, which create micro-edges, are produced on its surface. On the one hand, when the workpiece material is ground by this grit type, the undeformed chip thickness decreases dramatically due to the increase in the micro-cutting edges, which allow for hard and brittle materials to be removed mainly in a plastic form, reducing the subsurface damage caused by stress concentration [9,10]. On the other hand, the grit produces microporous fragmentation during the grinding process, forming new micro-edges, which improve the grinding efficiency. Therefore, the P-diamond grit has a broad application prospect in the highly efficient and low-damage grinding of hard and brittle materials.

Several studies have been carried out to reveal the generation mechanism of the pore on the grit, considering the superior material properties of P-diamond grit. Ohashi et al. [11], Mehedi et al. [12], and Takasu et al. [13] focused on studying how to use thermochemical corrosion technology to prepare P-diamond grits. Finally, the diamond surface was corroded in a mixed high-temperature gas flow environment of hydrogen and nitrogen, and P-diamond abrasives were successfully prepared. Wang Junsha et al. [14,15,16] also used corrosion methods to compare the differences in corrosion degree and morphology of diamond {100} and {111} crystal planes caused by corrosion agents such as Fe, Co, and Ni. They further explored and optimized the chemical reaction mechanism of these corrosion agents on M-diamond, thereby optimizing the corrosion process. Lee et al. [17] and Jeong et al. [18] used femtosecond laser technology to drill holes on the surface of diamond abrasive particles to prepare P-diamond abrasive particles. They also verified that femtosecond laser drilling on diamond produces good roundness, no microcracks, and no thermal damage.

Studies on P-diamond grit preparation have been greatly advanced, and the pore structure has less influence on the grits’ strength. Nevertheless, investigations on the material removal mechanism using P-diamond grits are still limited. Li et al. [19,20] used a mechanical model of stress distribution at the interface between the surface modified layer and the matrix of brittle materials, providing a theoretical basis for optimizing the toughness domain grinding process of brittle materials. Mao et al. [21] used a toughness domain critical grinding depth model for hard and brittle materials, indicating that at the toughness domain grinding scale, the surface roughness and damage depth of hard and brittle materials were significantly reduced. Zhou et al. [22] established a two-dimensional finite element model for the single-abrasive grain grinding of silicon carbide and analyzed the influence of the grinding edge radius and grinding wheel speed on the formation of silicon carbide chips. Numerous scholars at home and abroad have used single-abrasive particle scratch experiments to validate the above model and support its effectiveness. Guo et al. [23] and Rasim et al. [24] derived a quantitative chip-forming model and explored the effect of different rake angles of abrasive particles on material removal forms. The study showed that the size of the rake angle of abrasive particles significantly affects the ductile/brittle removal transformation of the material. Jin et al. [25] found through single-abrasive scratch experiments on fused quartz glass that only by reducing the scratch depth can crack formation be avoided. Based on this, a new method for predicting the surface quality of brittle material processing was proposed by combining the chip thickness model with finite element simulation. These research models were simulated using the finite element model. However, mesh distortion easily occurred during the finite element simulation process, leading to the computation’s convergence, especially in the grinding process. In addition, the simulation method cannot sufficiently clarify the physical phenomena in microscale processing. Therefore, studies on the material removal mechanism of SiC ceramics with P-diamond grit still remain in the bottleneck stage.

In recent years, the molecular dynamics (MD) method, which is essentially similar to the discrete element method (DEM), has been utilized to study the precision machining of brittle materials. Liu et al. [26] investigated the material removal behavior of SiC by diamond grit at the nanoscale by using MD. They found that plastic deformation and through-crystal fracture occurred in monocrystalline SiC. Meanwhile, Zhao et al. [27,28] and Li et al. [29] investigated the brittle/ductile transition conditions of silicon by MD. This method differs significantly from the finite element simulation method. It involves constructing a discrete unit ensemble based on the nature of the substance itself and then analyzing the physical behavior from a micro or macro perspective. This approach provides explanations that cannot be achieved through finite element analysis. In addition, this method avoids mesh distortion and other issues that may arise during the calculation process.

Consequently, this paper proposes a discrete element simulation model for the P-diamond grit grinding of SiC hard and brittle materials. The model reveals the removal mechanism of the SiC material by P-diamond grit grinding at the microscopic scale and explores the influence laws of grinding parameters and pore parameters on material removal. At the same time, the model determines the difference in grinding performance between P-diamond abrasive and P-diamond grit, so as to provide a more scientific theoretical basis for optimizing the grinding parameters. The research results of this paper will lay a scientific foundation for the efficient and low-damage grinding of hard and brittle materials represented by SiC ceramics, which has important theoretical significance and practical value.

## 2. Grinding Model

### 2.1. Model of SiC Ceramics by DEM

SiC ceramics are typically brittle materials that are difficult to machine, and their mechanical properties are similar to those of diamond. In this paper, Altair EDEM 2021 (Altair, Baltimore, MD, USA) was used to construct a physical model for grinding SiC ceramics with P-diamond grit grains. The SiC model was designed as a rectangular block 80 μm × 40 μm × 30 μm, and more than 97,000 particle cells were generated. The boundary conditions of the remaining surfaces were fixed. The Hertz–Mindlin bonded contact model was used to associate each particle according to Equation (1) [30], as follows:

(1) $$F_{n}^{e}=-K_{nHz}h^{\frac{3}{2}}$$

where $F_{n}^{e}$ denotes the elastic contact force between two particles; h is the overlap value between adjacent particles, and h = −g. The contact stiffness parameter $K_{nHz}$ is calculated as follows:

(2) $$K_{nHz}=\frac{4}{3}E^{*}\sqrt{R^{*}}$$

where E* is the effective modulus of elasticity defined by the Young’s moduli E_i_ and E_j_ and Poisson’s ratios v_i_ and v_j_ of two contacting particles:

(3) $$\frac{1}{E^{*}}=\frac{1-v_{i}^{2}}{E_{i}}+\frac{1-v_{j}^{2}}{E_{j}}$$

and R* is the effective radius of the particle:

(4) $$R^{*}=\frac{1}{R_{i}}+\frac{1}{R_{j}}$$

**Figure 1:**
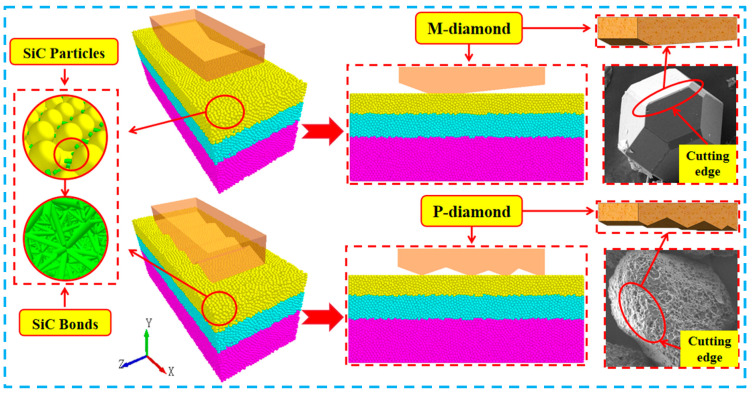
Model of SiC ceramics grinded by P-diamond grit.

The material properties, particle properties, and specific values involved in the model building process are listed in Table 1. The effect of the SiC discrete element model is displayed in Figure 1.

### 2.2. Model of Diamond Grit by DEM

The P-diamond grit is obtained using the etching process. Figure 1 illustrates that the M-diamond grit has a relatively smooth appearance and a small number of grinding edges. However, the surface of the P-diamond grit is rougher, with many grinding edges on the surface. In addition, the grits are typically attached to the grinding wheel’s substrate, moving circularly. The P-diamond grits are extended in the grit modeling given that the trajectory of each grit involved in grinding at the micrometer level is approximately a straight line. Thus, each grinding edge on its surface lies flat on a beveled surface. The height of the bevel is the tangential thickness of a single grit, and the depth of the cut of each grinding edge on the bevel should be incrementally increased. The rake angle of the grit is set at a constant value of 160°, to which the material properties of diamond are assigned. Subsequently, the M-diamond grits are modeled using the same approach. The material properties and specific values involved in the modeling process of P-diamond and M-diamond grits are listed in Table 2, and the schematic of the DEM model of porous diamond grinding SiC ceramics is shown in Figure 1.

### 2.3. Simulation Program

The grinding performance of P-diamond grit is affected by three key factors: cutting edge density, grinding wheel speed, and undeformed chip thickness. The term “cutting edge density” is used to describe the number of cutting edges per unit of cutting thickness on the surface of the abrasive grain. The grinding wheel speed is defined as the linear speed of the grinding wheel. Undeformed chip thickness refers to the thickness of a single cut of each abrasive grain when cutting between the grinding wheel and the workpiece. These parameters are of great significance in the grinding process, as they have a notable impact on the quality of the workpiece surface, its roughness, and the extent of damage to the workpiece. For each grinding factor, three groups of experiments were conducted using the controlled experiment method and the control variable method. The effects of cutting edge density, grinding wheel speed, and undeformed chip thickness on the material removal of SiC ceramics by P-diamond grit were investigated, considering the amount of damage to the SiC bonds, the change in the energy of the SiC particles, and the magnitude of the total grinding force as the characterizing quantities. During the simulation process, the diamond grit model grinds the surface of the SiC model based on the predetermined scheme, retaining factors that affect the grinding of SiC ceramics, such as the grinding angle. Table 3 shows the specific simulation scheme.

## 3. Results and Discussion

### 3.1. Effect of Micro-Cutting Edge Density on Material Removal Process of SiC Ceramics

**Figure 2:**
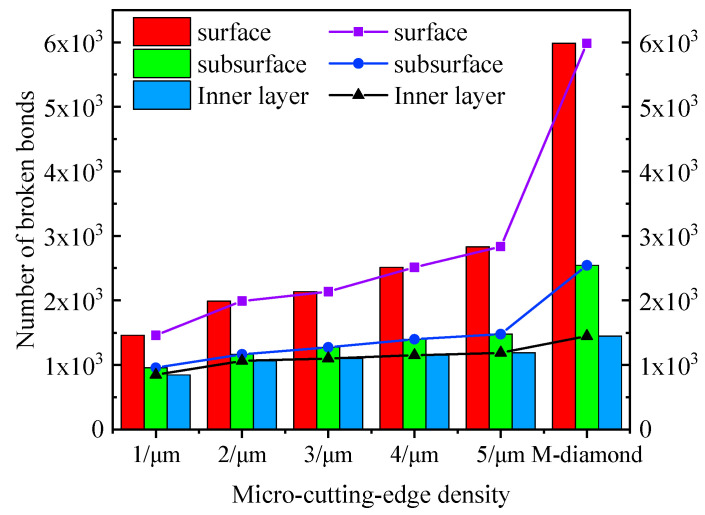
Number of broken bonds in each SiC layer at stages of scratch and plowing.

**Figure 3:**
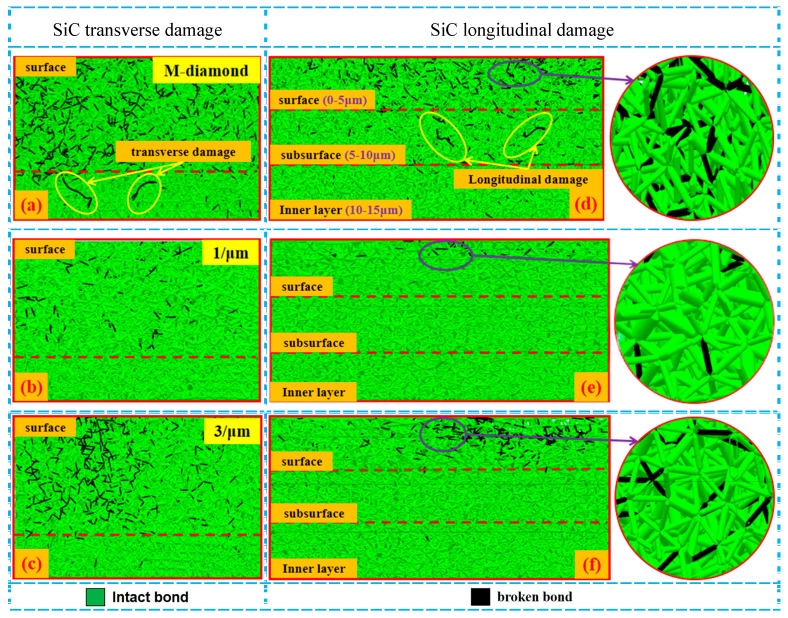
Distribution of broken bond conditions in each SiC layer at scratch plowing stages: (**a**–**c**) represent the transverse bond damage in the surface layer of SiC after diamond grit grinding with different cutting—edge densities. (**d**–**f**) indicate the longitudinal damage of bonding bonds in each layer of SiC.

Generally, the grinding process is mainly composed of four stages: scratch, plowing, chip formation, and grit detachment. Figure 2 displays the changes in the number of accumulated damages in each surface, subsurface, and inner layer of bonds when SiC ceramics are ground by diamond grits with different cutting edge densities during the scratch and plowing stages. The SiC surface with a depth of 5 μm is defined as the surface layer, a depth of 5–10 μm represents the subsurface layer, and a depth of 10–15 μm depicts the inner layer. This definition is extended to the following figure representations. When the M-diamond grit is utilized, more than 5.98 × 10^3^ bonds on the surface are damaged. The values of damaged bonds on the subsurface and inner layer are 2.55 × 10^3^ and 1.44 × 10^3^, respectively. By contrast, when the P-diamond grit is applied, the number of damaged bonds in all layers of SiC is lower than that of M-diamond grits. When the micro-cutting edge density is 1/μm, the number of damage bonds on the SiC surface layer, subsurface layer, and inner layer is 1.46 × 10^3^, 0.95 × 10^3^, and 0.85 × 10^3^, respectively. As the micro-cutting edge density increases gradually to 5/um, the number of damaged bonds on each layer slightly increases. However, the number remains significantly lower than that of the M-diamond grit. Figure 3 shows the distribution of damage to the SiC model at the scratch and plowing stages for P-diamond with different cutting edge densities and M-diamond grits. Evidently, the damage areas are mainly concentrated on the surface layer with a tendency to extend downward and to the sides when ground by M-diamond grit. In addition, the damage is concentrated on the surface layer when ground by P-diamond grit, and the depth of damage is minimal (Figure 3b,c). Thus, the damage to the SiC bond is more pronounced when subjected to M-diamond grit. However, the effect of micro-cutting edge density on the damage to the SiC bond is small.

**Figure 4:**
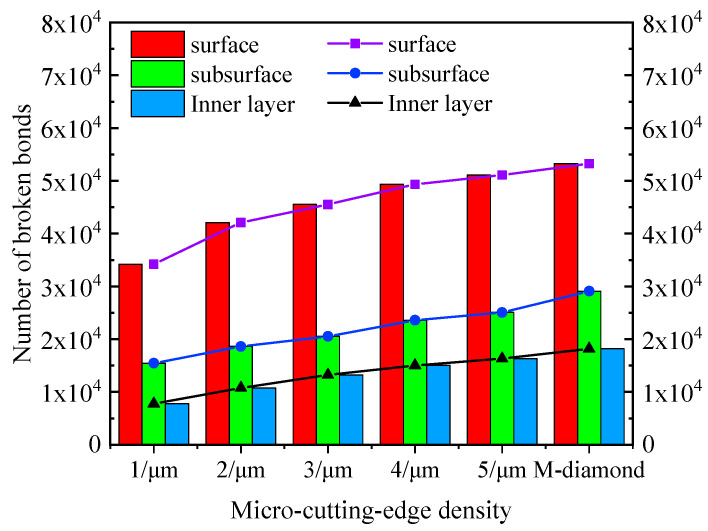
Number of cumulative broken bonds in each layer of SiC at stage of chip formation.

**Figure 5:**
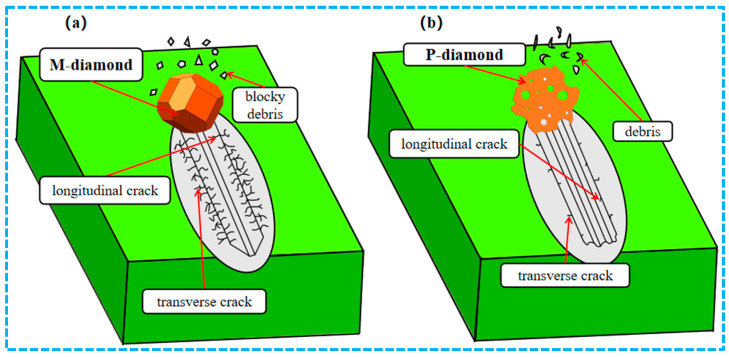
SiC removal mechanism for diamond grit grinding: (**a**) M-diamond, (**b**) P-diamond.

**Figure 6:**
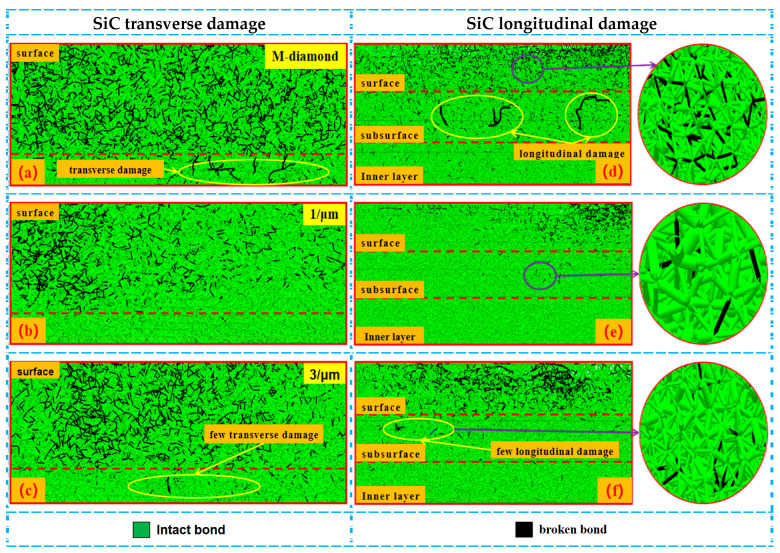
Distribution of cumulative damage conditions of bonding bonds in each layer of SiC at chip formation stage: (**a**–**c**) represent the transverse bond damage in the surface layer of SiC after diamond grit grinding with different cutting—edge densities. (**d**–**f**) indicate the longitudinal damage of bonding bonds in each layer of SiC.

During the chip formation stage, as the grit gradually cuts into the SiC surface and the chips are formed, the damage degree of SiC ceramics ground by M-diamond or P-diamond grits further increases. Figure 4 shows that the number of damaged bonds on the surface increases to 5.33 × 10^4^ (88.78%) when M-diamond grit is applied. The number of damaged bonds on the subsurface and inner layers also increases to 2.91 × 10^4^ and 1.82 × 10^4^, respectively, compared with that at the scratch plowing stage. Figure 5 shows the SiC removal mechanism for diamond grit grinding, and Figure 6a shows the distribution of damage to SiC bonds during the grinding of the M-diamond grit under this stage. The depth of the damaged area on SiC gradually extends from the surface layer to the subsurface and inner layers. Moreover, a breakage bond extension phenomenon can be observed on both sides of the surface layer. The bond damage trend is consistent with the phenomenon in Figure 5. The damage area of SiC ground by P-diamond grit also increases gradually and becomes more apparent as the micro-cutting edge density increases. The surface, subsurface, and inner layer of SiC can sustain bond damage ranging from 3.42 × 10^4^ to 5.11 × 10^4^, 1.55 × 10^4^ to 2.51 × 10^4^, and 0.78 × 10^4^ to 1.63 × 10^4^, respectively (Figure 4). Compared with M-diamond, the maximum reduction in the number of damages to SiC bonds was 35.76%, 46.86%, and 57.31%, whereas the minimum reduction was 4.09%, 13.87%, and 10.17%. Figure 6b,c show that although the amount of bond damage in each layer of SiC increases slightly as the micro-cutting edge density increases, this damage remains primarily concentrated on the surface layer, with relatively short extension lengths of damaged bonds. Moreover, the overall damage extent is still less than that in the case of M-diamond grit grinding. At this stage, the SiC bond is significantly damaged when ground by M-diamond grit, and the damage depth continues to increase. The increase in micro-cutting edge density increases the damage on the SiC bond, but it is still mainly concentrated in the surface layer, resulting in a more stable grinding process.

**Figure 7:**
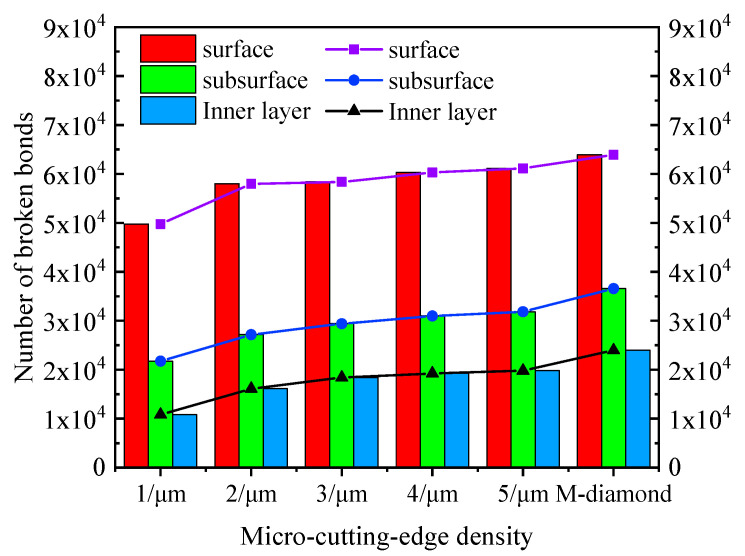
Number of cumulative broken bonds in each layer of SiC in grit detachment stage.

**Figure 8:**
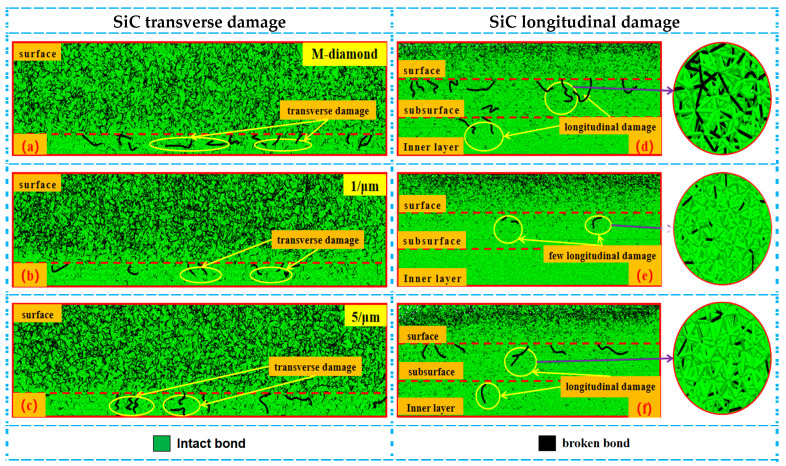
Distribution of cumulative damage conditions of bonds in each layer of SiC at grit detachment stage: (**a**–**c**) represent the transverse bond damage in the surface layer of SiC after diamond grit grinding with different cutting—edge densities. (**d**–**f**) indicate the longitudinal damage of bonding bonds in each layer of SiC.

During the detachment stage, the grit gradually cuts out of the SiC surface. The degree of damage by M-diamond and P-diamond grits to SiC bonds gradually tends to flatten. For M-diamond grit, the number of damage bonds on the SiC surface, subsurface, and inner layer is 6.39 × 10^4^, 3.66 × 10^4^, and 2.40 × 10^4^, respectively (Figure 7). However, when grinding using P-diamond grit, the damaged bond number ranges from as small as 4.97 × 10^4^, 2.17 × 10^4^, and 1.08 × 10^4^ to as large as 6.11 × 10^4^, 3.18 × 10^4^, and 1.98 × 10^4^, respectively. Compared with M-diamond grit, P-diamond grit damages bonds in the minimum percentage of 77.78%, 59.29%, and 45% and in the maximum percentage of 95.62%, 86.88%, and 82.5% at the same time and parameters. Figure 8a shows a large amount of visible damage to the SiC subsurface layer bonded to the inner layer during M-diamond grinding. Moreover, Figure 8b,c show that the damage to the SiC bond during P-diamond grinding is mainly concentrated on the surface layer, with a small amount of damage occurring in the subsurface layer. Meanwhile, the damage in the subsurface layer increases as the micro-cutting edge density increases. However, the damage is not evident on the inner layer. When the density of the micro-cutting edge reaches 5/μm, the material removal performance of P-diamond is similar to that of M-diamond. The damage to the SiC bond caused by M-diamond grit is significant and extends throughout the entire process. The damaged depth and concentration are noticeable. On the contrary, the damage degree of SiC ground by P-diamond grit is minimal, and the damaged depth and area are mainly concentrated on the surface layer of SiC. Few instances are observed on the subsurface and inner layers. However, with the increase in micro-cutting edge density, the number of SiC damaged bonds increases simultaneously.

The removal capacity of diamond grit for SiC ceramics can be characterized by the amount of damaged bonds. The grinding process of P-diamond grit is “gentler,” whereas that of M-diamond grit is more “vigorous” because the surface of the P-diamond grit has numerous micro-cutting edges with a different undeformed chip thickness. During the scratch plowing stage, the SiC surface is initially contacted by the cutting edge of the P-diamond grit with small undeformed chip thickness. The bond of the SiC surface layer gradually breaks under the grinding force, without causing subsurface or inner layer damage. During the chip forming stage, micro-cutting edges on the grit surface participate in grinding as the grit deepens. SiC bonds continue to break, causing damage primarily on the surface layer. This damage gradually extends to subsurface and inner layers as the density of micro-cutting edges increases, but the amount of damaged bonds remains small. During grit detachment, smaller undeformed chip thickness micro-cutting edges detach from the SiC surface, while larger ones maintain contact. Additionally, significant grinding residual stress persists on the SiC surface layer, resulting in gradual surface-focused SiC fracture. Additionally, at increased cutting edge densities, the damage to SiC layer bonding gradually approximates that caused by M-diamond grit grinding. P-diamond grit improves the grinding of SiC, reducing subsurface and inner layer damage compared to M-diamond grit. This is due to reduced damage from bonds on SiC layers. However, its effectiveness decreases with increasing micro-cutting edge density. Therefore, P-diamond grit’s micro-cutting edge density should be controlled at 1–3/μm.

**Figure 9:**
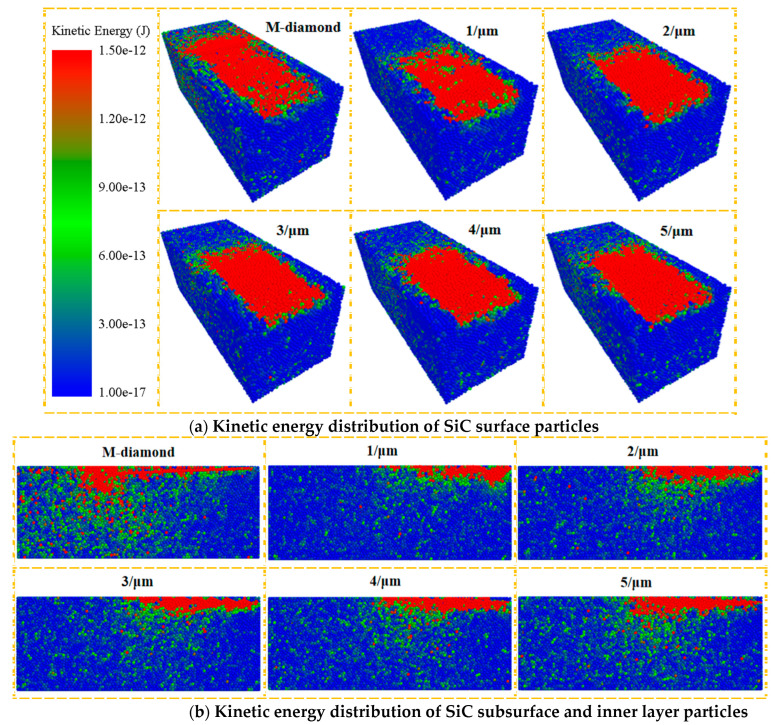
Distribution of kinetic energy of particles in each layer when diamond grits with different cutting edge densities grind SiC at chip formation stage.

The breakage of SiC bonds is caused by the kinetic energy of SiC particles when subjected to the grinding force of diamond grit. This energy causes SiC particles to break free from each other, resulting in bond breakage. The kinetic energy of SiC particles and the severity of the bond breakage increase with proximity to the surface of the diamond grit. Chip formation produces the most concentrated SiC damage. A significant difference in the kinetic energy of SiC particles during the grinding process is observed between M-diamond and P-diamond grit with different micro-cutting edge densities. Figure 9a illustrates that during the grinding of SiC with M-diamond grit, a considerable number of active particles are present in the SiC surface layer. These particles are distributed throughout the grinding area, with their kinetic energy decreasing from the grinding zone to the two sides. This results in a noticeable delayed extension phenomenon. However, when grinding SiC with P-diamond grit, the number of active particles on the surface layer is limited, and the distribution of these particles is relatively concentrated, with less extension to the sides. However, as the density of the grinding edge increases, the number of active particles in the surface layer of SiC gradually increases, and the degree of elongation and dispersion slowly increases. This phenomenon is also supported by the data presented in Figure 9b. The extent of SiC damaged bonds incurred during the grinding process is directly proportional to the kinetic energy of the SiC particles in each layer. This energy is, in turn, related to the magnitude of the grinding force applied to the SiC particles. The undeformed chip thickness of M-diamond grit is larger than that of P-diamond grit. Furthermore, the grinding force generated on the SiC particles affects a wide range. The P-diamond grit is characterized by the presence of numerous surface pores, which results in a relatively small undeformed chip thickness. Consequently, the grinding force generated on SiC particles is diminished, and the impact range is concentrated. Consequently, the kinetic energy of the SiC particles ground by P-diamond grit can be significantly reduced, as well as the range of energy transfer. However, the effect intensifies as the density of the micro-cutting edge increases, thereby reducing the stability of the P-diamond grit. When SiC particles are subjected to grit with higher kinetic energy and a wider energy transfer range, there is a greater likelihood of the SiC bond experiencing centralized breakage and extension. Furthermore, the grinding performance of the grit becomes increasingly unstable, resulting in more severe damage to the material. In conclusion, the overall grinding performance of P-diamond grit is more stable, significantly reducing the propensity of SiC subsurface and inner layer bond damage during the grinding process. Nevertheless, the effect diminishes with increasing the density of the cutting edge. Nevertheless, it still possesses certain advantages over M-diamond grit.

### 3.2. The Effect of Grinding Wheel Speed on the Material Removal Process of SiC Ceramics

**Figure 10:**
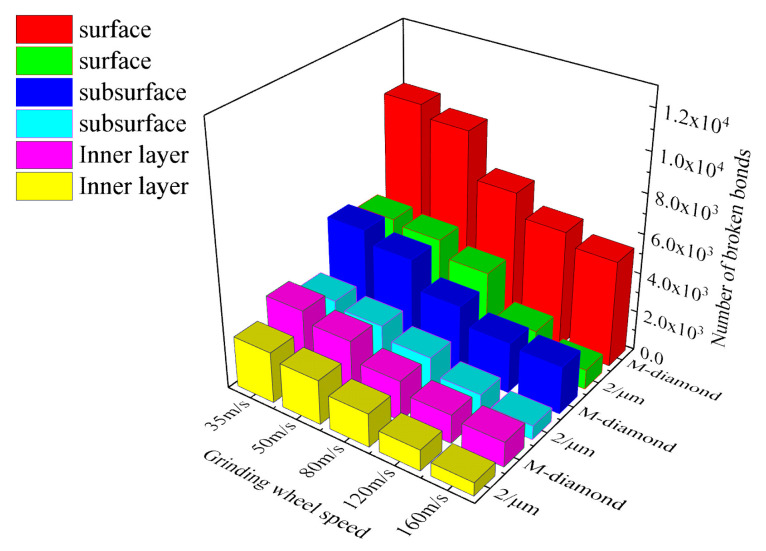
Number of broken bonds in each layer of SiC at scratch plowing stage.

**Figure 11:**
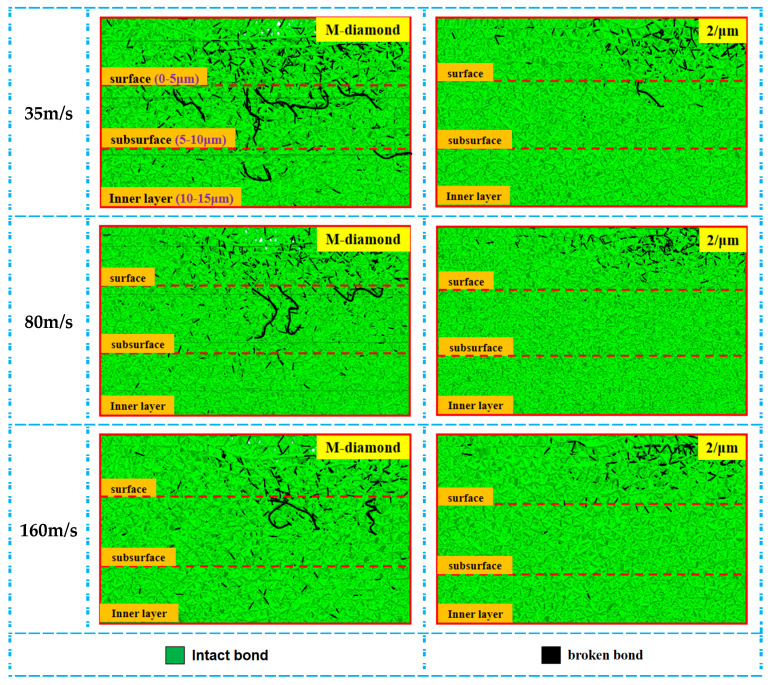
Distribution of cumulative damage conditions of bonds in each SiC layer at scratch plowing stage.

The grinding wheel speed is one of the most important grinding parameters that affect the material removal process. Figure 10 shows the statistics of changes in the number of accumulated damages in each surface, subsurface, and inner bond layer when ground by M-diamond and P-diamond grits with a cutting edge density of 2/μm at the scratch plowing stage. The amount of damaged bonds in each layer ground by M-diamond grit decreases as the grinding wheel speed increases. When the grinding wheel speed is 35 m/s, the number of damaged bonds is 9.99 × 10^3^ on the surface, 5.49 × 10^3^ on the subsurface, and 3.53 × 10^3^ on the inner layer. When the grinding wheel speed is increased to 160 m/s, the number of damaged bonds in each layer decreases to 5.38 × 10^3^, 2.39 × 10^3^, and 1.26 × 10^3^. However, when the P-diamond grit is utilized, the amount of damaged bonds on each layer is considerably less than that of M-diamond grit. When the grinding wheel speed is 35 m/s, the amount of damaged bonds on each layer is 5.06 × 10^3^, 2.91 × 10^3^, and 2.57 × 10^3^. When the grinding wheel speed reaches 160 m/s, the number of damaged bonds decreases to 1.0 × 10^3^, 0.71 × 10^3^, and 0.67 × 10^3^. Figure 11 shows a cloud diagram of the distribution of damage conditions in each layer when M-diamond grit and P-diamond grit grinds SiC at different grinding wheel speeds at this stage. The damage trends of the bonds in various layers when grinding SiC with P-diamond grit at different grinding wheel speeds are similar to those of M-diamond grit. However, the damage depth is shallower than that of M-diamond. At this stage, the damage to the SiC bond is more significant when using M-diamond grinding at different speeds. The damage caused by P-grinding is slightly weaker, but the trend of bond damage with respect to grinding wheel speed remains the same for both methods.

**Figure 12:**
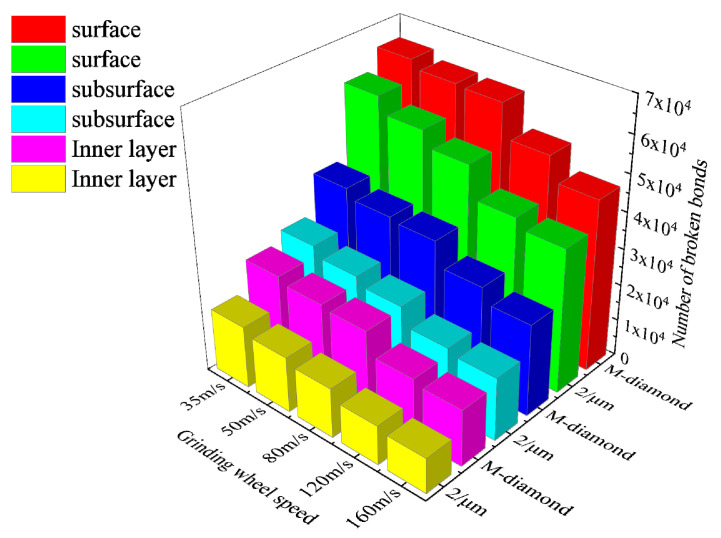
Number of broken bonds in each layer of SiC at stage of chip formation.

**Figure 13:**
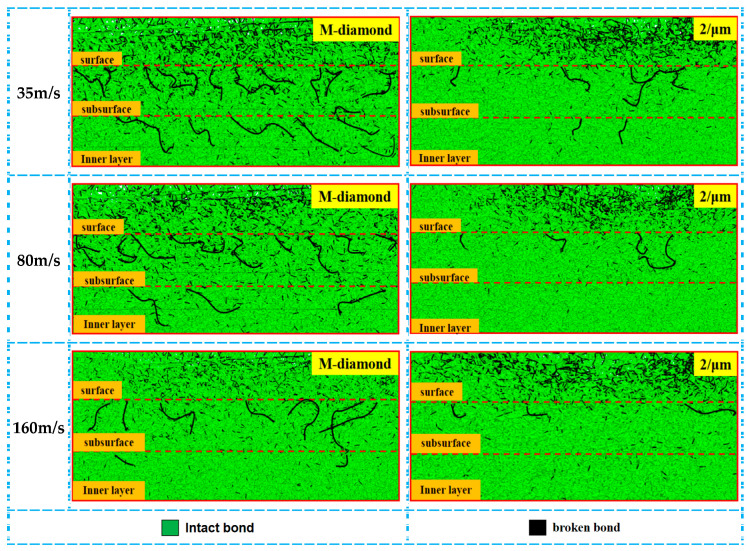
Cumulative damage distribution of bond bonds in SiC layers at different grinding wheel speeds during the chip formation stage.

During the chip formation stage, the influence of grinding wheel speed on the damage area of SiC bonds is further intensified, and the overall damage trend of the bonds is still consistent with that in the scratch plow stage. In addition, the damage to the SiC bond ground by M-diamond grit is more severe than that of P-diamond grit. Figure 12 shows the statistics of changes in the number of accumulated damages in each surface, subsurface, and inner bond layer when SiC is ground by M-diamond grit and P-diamond grit at different grinding wheel speeds. When the grinding wheel speed is 35 m/s, the accumulation of surface, subsurface, and inner bond damage in the case of M-diamond grit grinding reaches 6.46 × 10^4^, 3.91 × 10^4^, and 2.53 × 10^4^, respectively. In the case of P-diamond grit grinding, the accumulation of bond damage for each layer is 5.92 × 10^4^, 2.83 × 10^4^, and 1.71 × 10^4^. These values are 8.46%, 27.54%, and 32.61% lower than those of M-diamond. Figure 13 shows a cloud diagram of the distribution of damage conditions in each layer when M-diamond grit and P-diamond grit grind SiC at different grinding wheel speeds at this stage. The degree of damage to SiC by the two types of diamond grit is almost equal at a grinding wheel speed of 35 m/s. The amount of SiC bond damage is minimized when the grinding wheel speed is 160 m/s. The accumulation of bond damage in each SiC layer of ground by M-diamond grit decreases to 4.70 × 10^4^, 2.52 × 10^4^, and 1.55 × 10^4^. The depth of damage extends from the subsurface layer to the inner layer. In the case of P-diamond grit grinding, the accumulation of bond damage in each layer decreases to 3.96 × 10^4^, 1.73 × 10^4^, and 0.97 × 10^4^, which is 15.75%, 31.49%, and 37.46% lower than that of M-diamond, respectively, and the depth of the damage is mainly concentrated on the surface layer. Figure 12 also shows that the material damage of SiC is severer when the grinding wheel speed is in the range of 35 m/s to 80 m/s. For the case of M-diamond grit, the extent of bond damage in each layer of SiC decreases by 3.98%, 8.72%, and 9.47%. By contrast, for the P-diamond grinding case, where the counterpart decreases by 14.34%, 16.83%, and 20.09%, the decreasing trend of damage is moderate. By increasing the grinding wheel speed from 80 m/s to 160 m/s, the amount of bond damage of each SiC layer ground by M-diamond grit can be reduced by 24.36%, 29.28%, and 32.45%, respectively. However, in the case of P-diamond grinding, the decrease is 21.96%, 26.6%, and 28.98%, a significant decrease in the bond damage trend. Therefore, to ensure the grinding performance of diamond grit, the critical grinding wheel speed is set to approximately 80 m/s. The tendency for bond damage decreases with increasing grinding wheel speed but is still more significantly affected by M-diamond grinding. For SiC grinding, a grinding wheel speed of more than 80 m/s is slightly more favorable.

**Figure 14:**
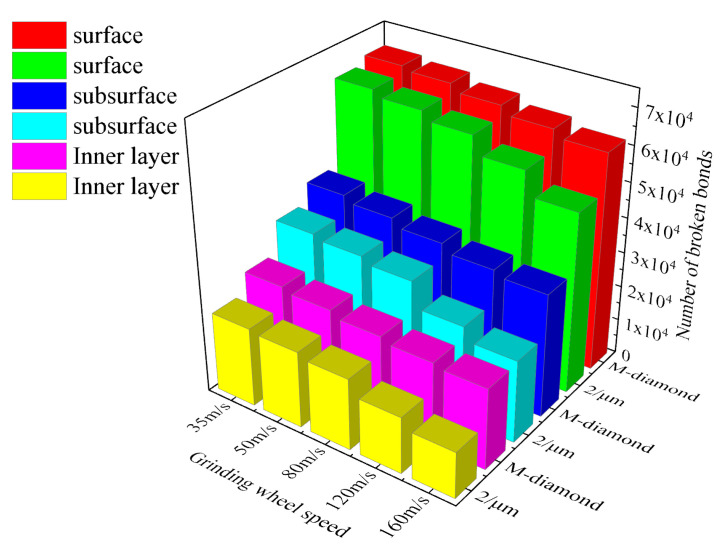
Number of broken bonds in each layer of SiC at stage of grit detachment.

**Figure 15:**
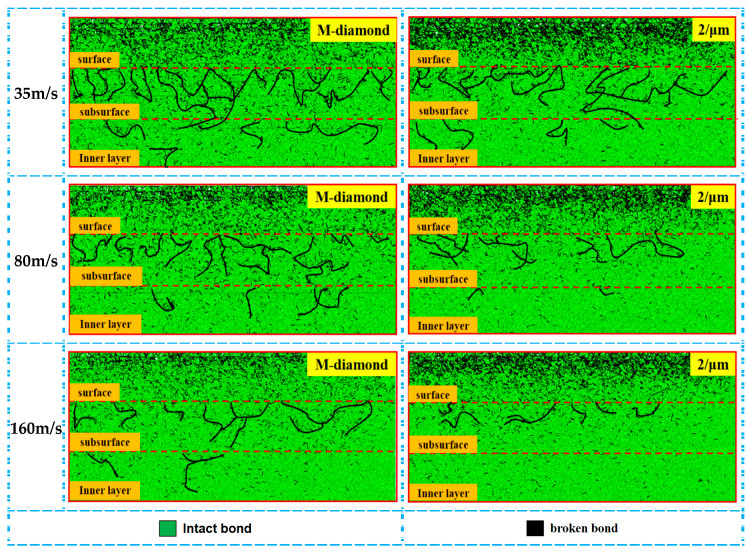
Distribution of cumulative damage conditions of bonds in each SiC layer at grit detachment stage.

During the grit detachment stage, the damage to the SiC bonds caused by both diamond grits gradually levels off. However, the amount of damage to the bonds increases slightly due to the grinding edges of the P-diamond surfaces still acting on the SiC surfaces. Nevertheless, the damage is much less than that caused by M-diamonds after grinding. Figure 14 shows the statistical changes in the number of accumulated damages in each surface, subsurface, and inner bond layer when the M-diamond grit and P-diamond grit grind SiC at different grinding wheel speeds at the grit detachment stage. When the grinding wheel speed is 35 m/s, the cumulative damages to the bonds of each layer of SiC at the end of M-diamond grinding are quantified as 6.92 × 10^4^, 4.26 × 10^4^, and 2.81 × 10^4^. By contrast, the cumulative bond damages in each SiC layer at the end of P-diamond grinding are 6.71 × 10^4^, 3.71 × 10^4^, and 2.25 × 10^4^. Figure 15 shows a cloud diagram of the distribution of damage conditions in each layer when M-diamond grit and P-diamond grit grind SiC at different grinding wheel speeds at this stage. Although the amount of bond damage is less in the latter case, the overall damage to the SiC bond is severe for both diamond grits at low speeds. When the grinding wheel speed increases to 80 m/s, the cumulative bond damages of each SiC layer after M-diamond grinding are 6.61 × 10^4^, 3.88 × 10^4^, and 2.53 × 10^4^. Moreover, the cumulative bond damages in each SiC layer at the end of P-diamond grinding are 6.29 × 10^4^, 3.43 × 10^4^, and 2.04 × 10^4^, which decreases by 4.81%, 11.65%, and 19.47% compared with the former values. The degree of SiC bond damage becomes significantly less for P-diamond than for M-diamond at this speed. When the grinding wheel speed is increased to 160 m/s, the cumulative bond damages in each SiC layer after M-diamond grinding are 6.19 × 10^4^, 3.51 × 10^4^, and 2.31 × 10^4^. Moreover, the cumulative damage of the bond of each SiC layer at the end of P-diamond grinding is 5.13 × 10^4^, 2.35 × 10^4^, and 1.31 × 10^4^. Compared with the M-diamond grit, it decreases by 17.08%, 33.22%, and 42.80%, exhibiting remarkable reduction and low damage. At this grinding wheel speed, P-diamond demonstrates superior grinding performance to M-diamond. Furthermore, the damage depth is concentrated on the surface layer of SiC.

**Figure 16:**
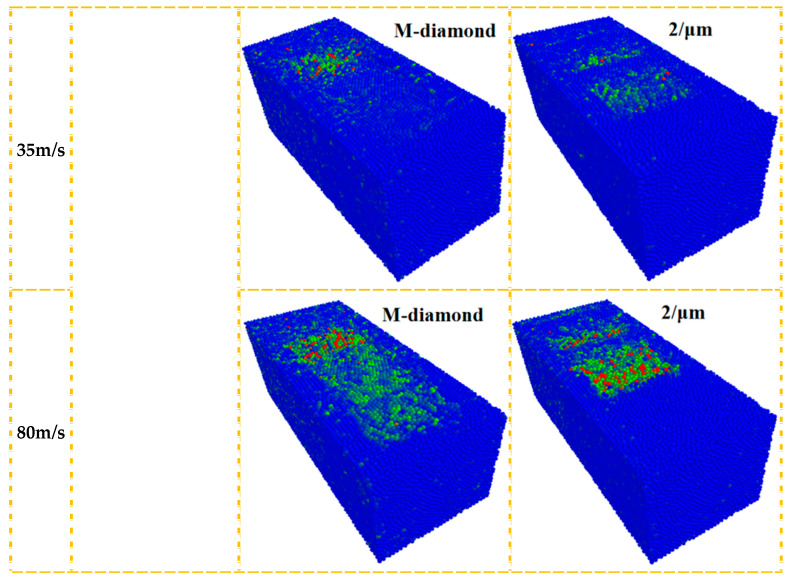

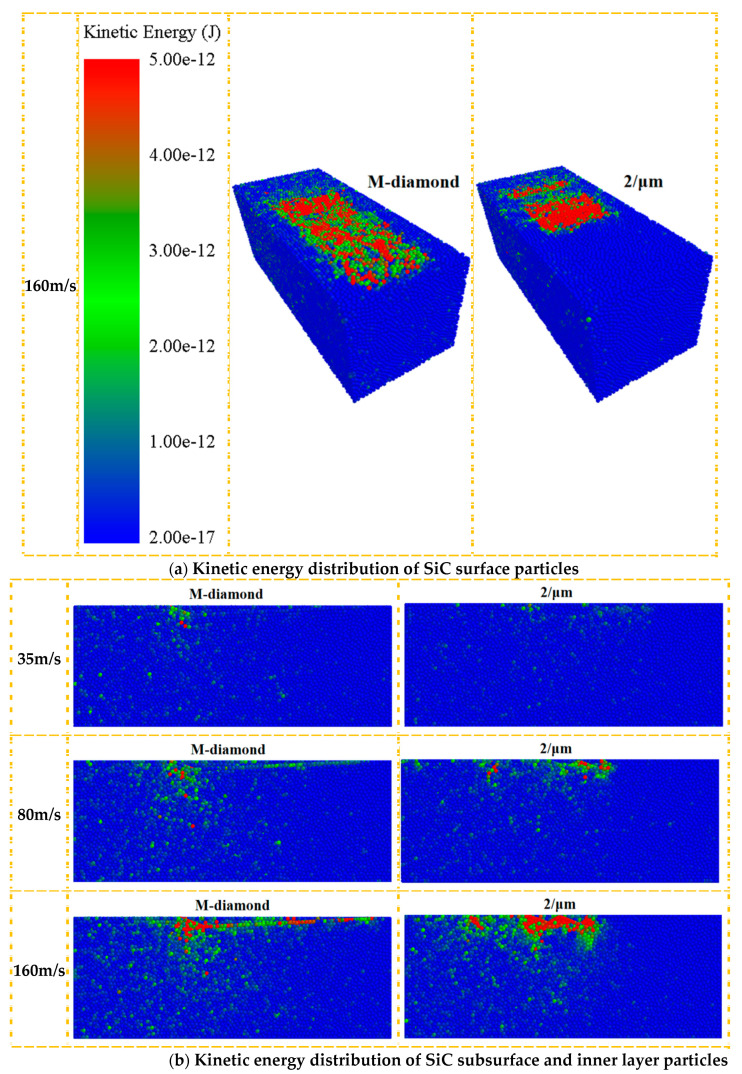
Distribution of kinetic energy of particles in each layer when diamond grit with different grinding wheel speeds grinds SiC at chip formation stage.

**Figure 17:**
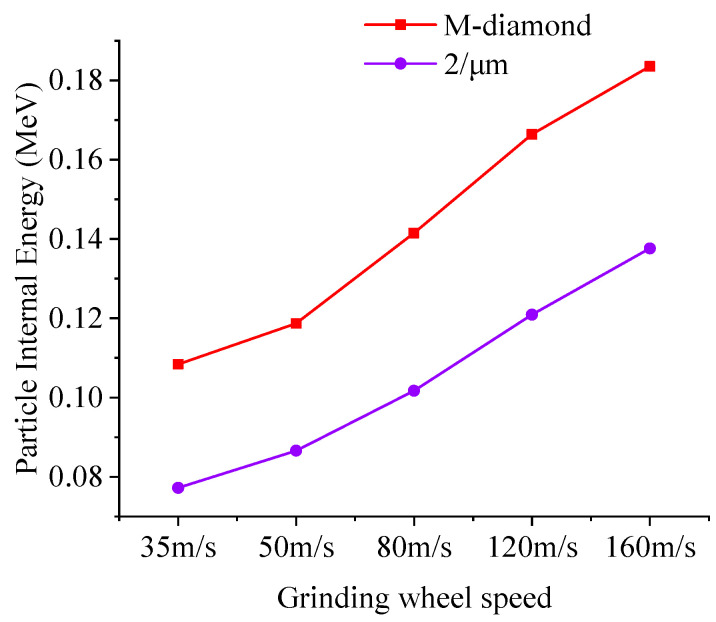
Variation in the average internal energy in the SiC surface particles at different grinding speeds during the chip formation stage.

During grinding, diamond grit extrusion and shearing can lead to SiC particle collisions. Particle collisions and energy increase as the grinding wheel speed rises. This raises the SiC/diamond contact area temperature, softening it and reducing the tangential grinding force. This minimizes SiC bond damage. The normal grinding force remains constant. Overall, the SiC layer bond strength damage is reduced. Figure 16 demonstrates that as the grinding wheel speed increases, the concentration of active particles on the SiC surface increases, whereas the dispersion of active particles in the subsurface and inner layers gradually increases, enhancing the energy transfer effect. However, in M-diamond grit grinding, the number of active particles and the degree of dispersion of SiC layers are greater than those achieved with P-diamond grit grinding. Meanwhile, Figure 17 shows that the average internal energy contained in the SiC surface particles gradually increases as the grinding wheel speed increases. SiC particles after M-diamond grit grinding have a higher internal energy than P-diamond grit grinding. Tangential and normal grinding forces during the process are influenced by the M-diamond grit surface and cutting depth of larger edges, causing diverse effects. Despite higher kinetic and internal energies, no significant surface temperature concentration is observed, leading to poor softening in the contact area. However, the presence of a large range of active particles leads to deeper bond damage. Although the SiC particles are considerably influenced by the grinding edges on the surface of the P-diamond grit, the tangential and normal grinding forces are generally smaller, thereby reducing the impact range. This finding results from numerous grinding edges with varying cutting depths on the P-diamond surface. Consequently, the elevated kinetic and internal energy in SiC particles raises the temperature of the contact area, inducing a softening effect that minimizes damage to the SiC layer bonds. In brief, the speed of the grinding wheel has a notable influence on the efficiency of P-diamond grit grinding. Boosting the speed of the wheel enhances the stability of the grinding process. Therefore, the risk of causing harm to the SiC subsurface and inner layers is minimized during grinding.

### 3.3. Effect of Undeformed Chip Thickness on SiC Ceramic Removal

**Figure 18:**
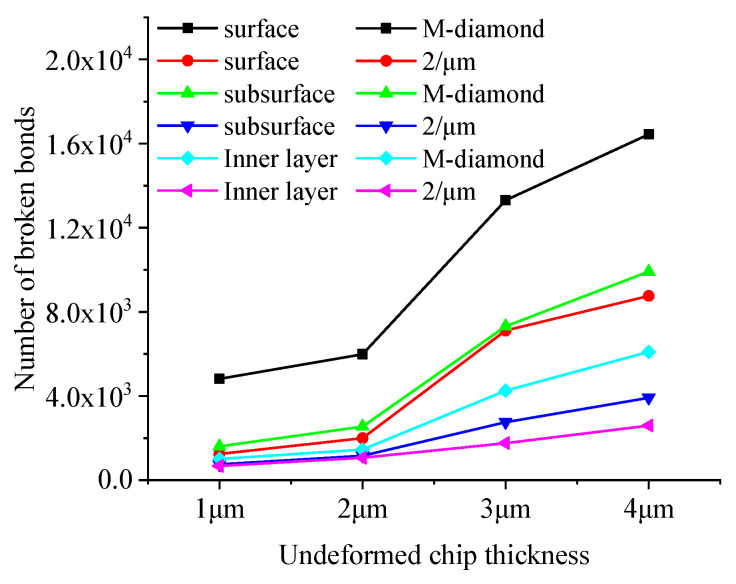
Number of broken bonds in each SiC layer at scratch plowing stage.

**Figure 19:**
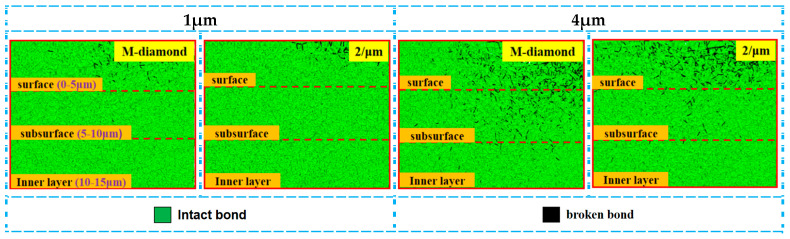
Distribution of cumulative damage conditions of bonds in each layer of SiC at scratch plowing stage.

The effect of undeformed chip thickness on the diamond grinding of SiC is significant, and the extent of diamond grit damage to SiC bonds varies with different grit thicknesses. Figure 18 shows the changes in the cumulative number of flaws in each surface, subsurface, and inner bond layer as M-diamond grit with different undeformed chip thicknesses and P-diamond grit with the micro-cutting edge density of 2/μm grind SiC using scratch plowing. During the scratch plowing stage, the bond damages in each layer of M-diamond grit used to grind SiC are 0.48 × 10^4^, 0.16 × 10^4^, and 0.10 × 10^4^ when the thickness of the undeformed chip is 1 μm. Meanwhile, the bond damages in each layer of P-diamond grit grinding are 0.12 × 10^4^, 0.075 × 10^4^, and 0.067 × 10^4^. When the undeformed chip thickness increases to 4 μm, the bond damages in each layer reach 1.64 × 10^4^, 0.99 × 10^4^, and 0.61 × 10^4^ for M-diamond grit grinding. Moreover, the bond damages in each layer reach 0.88 × 10^4^, 0.39 × 10^4^, and 0.26 × 10^4^ in P-diamond grit grinding. Figure 19 shows a cloud plot of the distribution of the damage condition of each layer when grinding SiC with M-diamond grit and P-diamond grit using different undeformed chip thicknesses. The quantity of SiC bond damages increases as the thickness of the undeformed chip increases. In addition, the number of bond damages increases significantly with the use of M-diamond grit grinding. Furthermore, the depth of bond damage in each SiC layer exhibits a linear increase. At this stage, M-diamond grinding causes more significant damage to the SiC bond at different undeformed chip thickness, whereas P-diamond grinding causes slightly weaker damage. However, the damage trends for both methods increase linearly with the increase in undeformed chip thickness.

**Figure 20:**
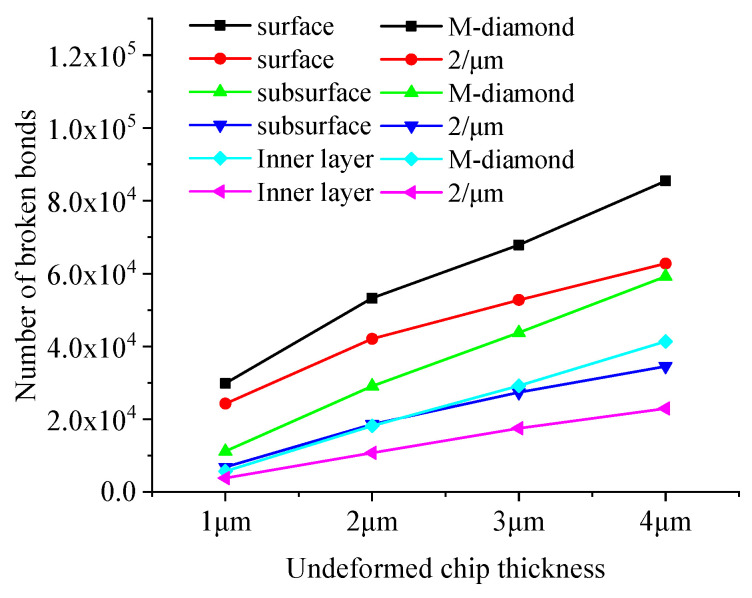
Number of broken bonds in each SiC layer at chip formation stage.

**Figure 21:**
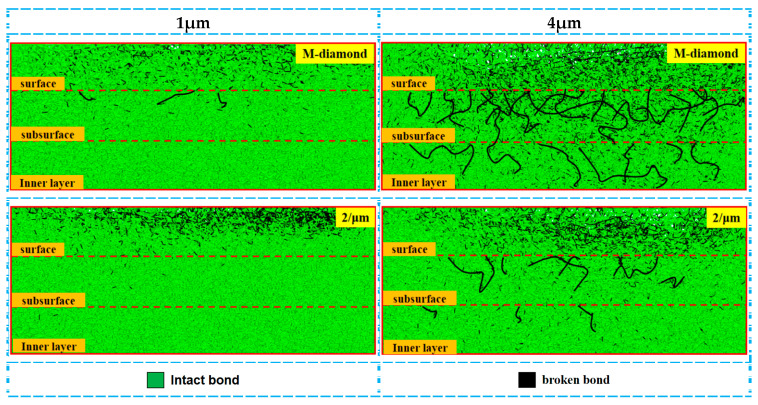
Distribution of cumulative damage conditions of bonds in each SiC layer at chip formation stage.

During the chip formation stage, the damage to the SiC bond increases substantially with the increase in the undeformed chip thickness. Figure 20 shows the changes in the cumulative number of flaws in each surface, subsurface, and inner bond layer upon SiC grinding by the M-diamond grit with different undeformed chip thickness and P-diamond grit with chip formation. When the undeformed chip thickness increases from 1 μm to 4 μm, the number of cumulative damages of superficial bonds increases from 2.97 × 10^4^ to 8.55 × 10^4^, that of subsurface bonds increases from 1.12 × 10^4^ to 5.92 × 10^4^, and that of inner bonds increases from 0.57 × 10^4^ to 4.14 × 10^4^ in the M-diamond grit grinding of SiC. By contrast, the amount of accumulated bond damage in each layer increases from 2.42 × 10^4^, 0.68 × 10^4^, and 0.38 × 10^4^ to 6.28 ×10^4^, 3.45 × 10^4^, and 2.30 × 10^4^, respectively, when the P-diamond grit was used to grind SiC. The average increase in SiC bond fracture in the case of M-diamond grinding is 77.43%, whereas that in the case of P-diamond grinding is 74.97%, indicating that the SiC bond damage by P-diamond grit is slightly affected by the undeformed chip thickness. Figure 21 shows a cloud plot of the distribution of the damage condition of each layer when grinding SiC with M-diamond grit and P-diamond grit using different undeformed chip thicknesses at this stage. As the undeformed chip thickness increases, the depth of bond damage in each SiC layer increases linearly. The depth of damage is shallow when the undeformed chip thickness is small. In the case of M-diamond grit grinding, the bond damage depth extends to the SiC subsurface layer, whereas the damage depth is concentrated on the surface layer in P-diamond grinding. When a single grit has a larger cutting thickness, the depth of SiC bond damage in both types of diamond grit grinding is extended to the subsurface layer or even the inner layer. However, in P-diamond grit grinding, the depth of damage is slightly smaller. The SiC bond damage at this stage is significantly affected by the undeformed chip thickness, but P-diamond grits can significantly reduce the large bond break caused by the increased undeformed chip thickness. The optimum undeformed chip thickness should be between 1 and 2 μm to achieve less damage.

**Figure 22:**
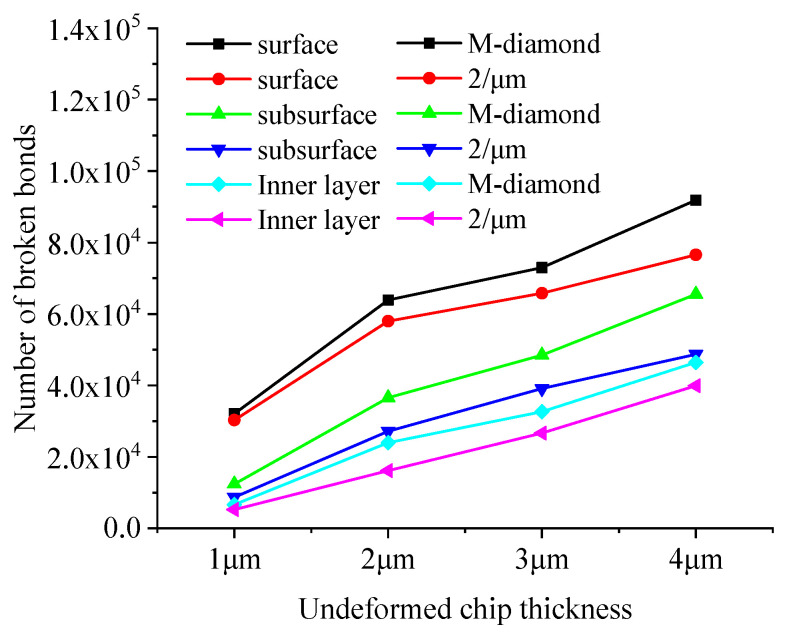
Number of broken bonds in each SiC layer at stage of grit detachment.

**Figure 23:**
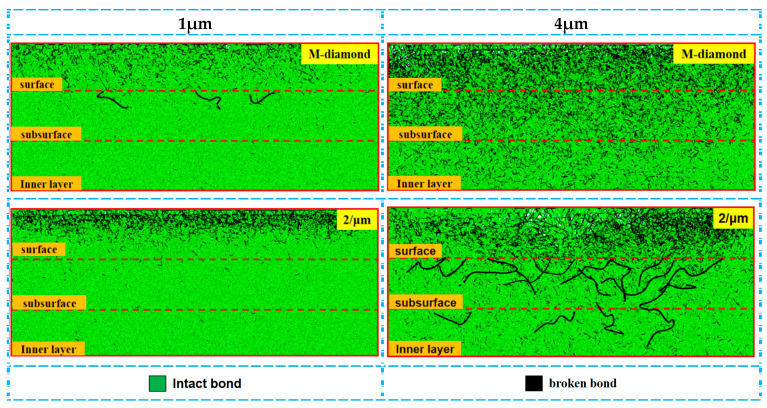
Distribution of cumulative damage conditions of bonds at grit detachment stage.

During the grit detachment phase, the degree of SiC bond disruption by both diamond grits gradually levels off, but the undeformed chip thickness continues to increase. Figure 22 shows the changes in the cumulative number of flaws in each surface, subsurface, and inner bond layer under M-diamond grit with different undeformed chip thickness and P-diamond grit grinding SiC with grit detachment. When the undeformed chip thickness is 1 μm, the cumulative damage quantities of bond in each SiC layer at the end of M-diamond grit grinding are finally 3.21 × 10^4^, 1.24 × 10^4^, and 0.66 × 10^4^ and 3.03 × 10^4^, 0.87 × 10^4^, and 0.52 × 10^4^ for P-diamond grit grinding, indicating a decrease by 5.21%, 29.63%, and 20.75% year-on-year. When the undeformed chip thickness increases to 4 μm, the cumulative damage quantities of the bond in each SiC layer at the end of M-diamond grit grinding are 9.19 × 10^4^, 6.55 × 10^4^, and 4.65 × 10^4^ and 7.66 × 10^4^, 4.87 × 10^4^, and 3.99 × 10^4^ for P-diamond grit grinding, exhibiting a decrease by 16.63%, 25.67%, and 14.11% year-on-year. Figure 23 shows a cloud plot of the distribution of the damage condition of each layer when grinding SiC with M-diamond and P-diamond grits under different undeformed chip thicknesses at this stage. When the undeformed chip thickness is 1 μm, the depth of the SiC bond damage extends to the subsurface layer after M-diamond grinding. By contrast, the depth of bond damage after P-diamond grinding is still concentrated on the surface layer. When the undeformed chip thickness is 4 μm, both diamond types experience more severe SiC bond damage during grinding. M-diamond grinding results in a greater depth of bond damage, reaching the inner layer and causing a large number of broken bonds. P-diamond, on the contrary, experiences shallower bond damage. In summary, using a smaller undeformed chip thickness can reduce the SiC bond damage and its depth. However, larger undeformed chip thickness can exacerbate bond damage, which can be severe regardless of the diamond grit type.

**Figure 24:**
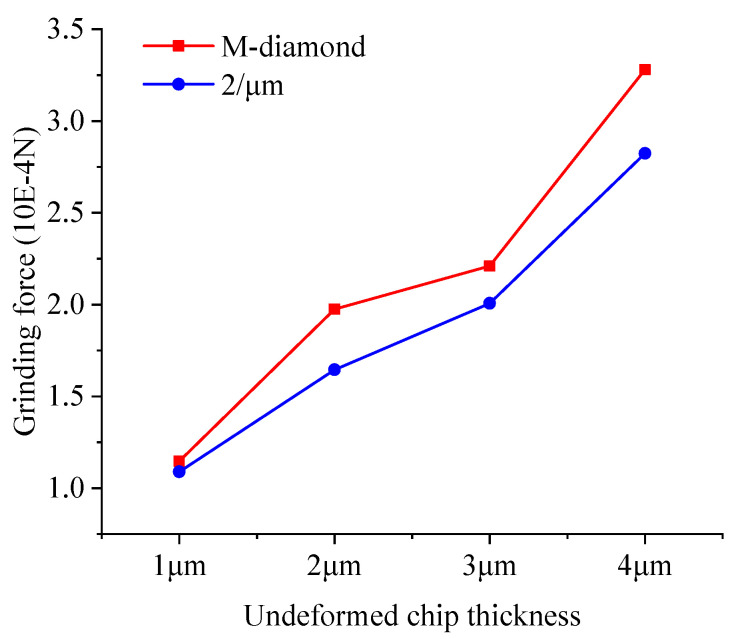
Variation in total grinding forces on SiC during diamond grit grinding with different undeformed chip thicknesses at the chip formation stage.

**Figure 25:**
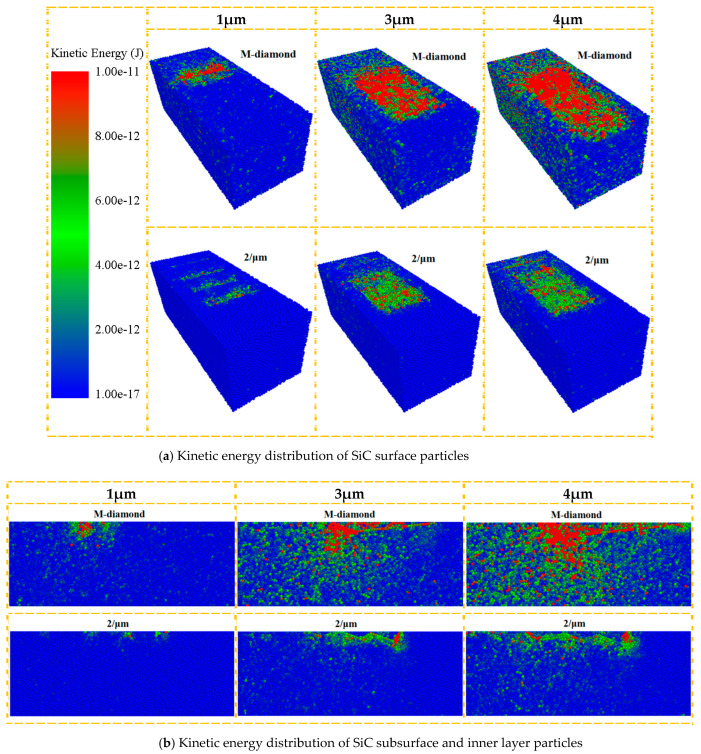
Distribution of kinetic energy of particles in each layer when diamond grit with different undeformed chip thicknesses grinds SiC at chip formation stage.

The effective area of the grinding edge on the diamond grit’s surface interacting with the SiC particles also increases as the undeformed chip thickness increases. This phenomenon leads to intensified collisions between the particles and elevated kinetic energy, resulting in a rapid increase in the amount of damage to the bonds. Figure 24 shows the variation in the total grinding force exerted on SiC when grinding with M-diamond grit and P-diamond grit with different undeformed chip thicknesses at the chip formation stage. The data show that the total grinding force increases linearly with the undeformed chip thickness. However, when grinding SiC, the grinding force generated by the P-diamond grit is significantly lower than that generated by the M-diamond grit. The grinding force magnitude varies and affects the SiC particles differently. Figure 25 shows a cloud diagram of the magnitude distribution of the kinetic energy of the particles in the different SiC layers when grinding with the two grit types in the corresponding case. The impact of M-diamond grit on SiC particles increases step by step with the undeformed chip thickness. This condition intensifies the energy transfer effect and linearly elevates the number and distribution of active particles in each layer, substantially increasing the amount of bond damage. The P-diamond grit, on the contrary, generates less grinding force than the M-diamond grit despite the increased effect under different undeformed chip thickness conditions. Thus, the number of active particles is reduced, the distribution is more concentrated, and the amount of bond damage is less. In summary, the effect of undeformed chip thickness on the grinding performance of the two diamond grits is significant. P-diamond, compared with M-diamond, greatly reduces the grinding force and damage to SiC when the undeformed chip thickness is small, resulting in more stable grinding. However, when the condition of undeformed chip thickness is large, the tendency of SiC to incur large area damage increases.

## 4. Conclusions

In this paper, a discrete element simulation model of SiC ceramic material grinding by P-diamond grit grains is established by the DEM. The controlled experimental simulation method is used to compare with M-diamond grit grains to reveal the mechanism of removing SiC ceramic materials by P-diamond grit grains from a microscopic point of view, and then the grinding parameters are optimized. In the future, this model can be employed to simulate and analyze the remaining grinding parameters (grinding angle, feed rate, etc.), as well as to further investigate the wear mechanism of porous diamond grits. The conclusions of this paper can provide new research ideas and a theoretical basis for the efficient and low-damage research of hard and brittle materials. The specific conclusions are as follows:

1. Compared to the more “aggressive” removal process of M-diamond grits, P-diamond grits are more “gentle” in removing SiC materials. Moreover, the damage to SiC ceramics caused by P-diamond grits is low, mainly concentrated in the surface layer of SiC. However, this damage degree increases with the increase in cutting edge density, so the optimal cutting edge density should be controlled within 1–3 μm.
2. Grinding SiC materials with P-diamond grits can cause workpiece damage due to the skin effect. The damage degree decreases with an increase in grinding wheel speed. When the grinding wheel speed exceeds 80 m/s, the removal of SiC during grinding becomes more efficient.
3. The results show that the undeformed chip thickness is very significant to the damage of SiC layers. In order to minimize the tendency and extent of such damage, the optimum undeformed chip thickness should be controlled between 1 and 2 μm.
4. Under reasonable grinding and pore parameters, P-diamond causes less damage to SiC materials than M-diamond. This finding indicates that P-diamond has better grinding performance than M-diamond.

## Figures and Tables

**Table 1:** Material property for SiC model [31,32,33,34,35].

Material Property	Value
Particle radius (μm)	0.5
Poisson rate (μ)	0.142
Density (kg/m^3^)	3215
Shear modulus (GPa)	192
Coefficient of restitution	0.5
Coefficient of static friction	0.5
Coefficient of rolling friction	0.01
Normal stiffness per unit area (kN/m^3^)	1.156 × 10^10^
Shear stiffness per unit area (kN/m^3^)	9 × 10^9^
Critical normal stress (MPa)	640
Critical shear stress (MPa)	270
Bonded disk radius (μm)	0.5

**Table 2:** Material property for diamond grit modeling [36,37,38].

Material Property	Value
Poisson rate (μ)	0.14
Density (kg/m^3^)	3500
Shear modulus (GPa)	700
Coefficient of restitution	0.5
Coefficient of static friction	0.5
Coefficient of rolling friction	0.01

**Table 3:** Simulation scheme for grinding SiC ceramics with P-diamond grit.

Parameter	Diamond Types	Cutting Edge Density (μm^−1^)	Grinding Wheel Speed (m/s)	Undeformed Chip Thickness (μm)
Cutting edge density	M-diamond	-	120	2
	P-diamond	1, 2, 3, 4, 5	120	2
Grinding wheel speed	M-diamond	-	35, 50, 80, 120, 160	2
	P-diamond	2	35, 50, 80, 120, 160	2
Undeformed chip thickness	M-diamond	-	120	1, 2, 3, 4
	P-diamond	2	120	1, 2, 3, 4

## Data Availability

The raw data supporting the conclusions of this article will be made available by the authors on request.
